# Preparation, Structural Elucidation, and In Silico Prebiotic Evaluation of Pectin Oligosaccharides from Apple Pectin

**DOI:** 10.3390/biom16101436

**Published:** 2026-10-02

**Authors:** Guoxiang Chi, Shuyu Zu, Yousi Fu, Huipeng Liu

**Affiliations:** 1 Xiamen Key Laboratory of Traditional Chinese Medicine Bio-Engineering, School of Pharmacy, Xiamen Medical College, Xiamen 361023, China; gxchi@xmmc.edu.cn; 2 Department of Chemical and Biochemical Engineering, College of Chemistry and Chemical Engineering, Xiamen University, Xiamen 361005, China; 3 Tianjin Key Laboratory of Food Science and Health, Research Institute of Public Health, School of Medicine, Nankai University, No. 38, Tongyan Road, Haihe Education Park, Tianjin 300350, China; 4 College of Chemistry and Chemical Engineering, Pingdingshan University, Pingdingshan 467000, China

**Keywords:** apple pectin, neutral pectin oligosaccharides, UPLC-HILIC-MS/MS, molecular simulation, colon fermentation simulation, prebiotic activity

## Abstract

Pectin oligosaccharides (POS) recovered from agro-industrial by-products are attracting attention as candidate prebiotics, yet obtaining structurally defined neutral POS and screening their activity remains challenging. This study prepared neutral POS from commercial apple pectin by controlled acid hydrolysis (0.05 M TFA, 80 °C, 1 h), ethanol precipitation, ultrafiltration (<3 kDa), and DEAE-Sepharose CL-6B chromatography, yielding a neutral P-1 fraction (3.6% *w*/*w*). The fraction was characterized by PMP-HPLC, HPSEC-RID, and UPLC-HILIC-MS/MS, revealing a bulk composition dominated by galactose, glucose, and arabinose, with five selected neutral oligosaccharides (DP 2–5). The prebiotic potential of these five selected, tentatively assigned oligosaccharides was evaluated in silico by simulation against 13 microbial glycosidases and by simulating colonic fermentation with CompuGUT using kinetic parameters derived from binding energies. The docking matrix showed favorable and enzyme-selective binding (−10.3 to −4.6 kcal/mol), with the strongest complexes involving component D–A7LXS9 (−8.9 kcal/mol) and component E–Q93GI5 (−10.3 kcal/mol). CompuGUT predicted complete hydrolysis of all five components within 48 h and increased mucosal biomass, indicating that these five selected compounds are predicted to be fermentable and to support microbial growth at the mucosal surface. These findings provide an integrated strategy for the preparation, structural characterization, and rapid functional screening of neutral POS, facilitating the identification of structurally defined pectin-derived prebiotic candidates.

## 1. Introduction

The gut microbiota sits between diet and host health, and its composition has been linked to both intestinal and systemic homeostasis [1]. Prebiotics, substrates selectively used by gut microbiota to confer a health benefit [2], give a dietary handle on this ecosystem: they typically enrich saccharolytic taxa such as *Bifidobacterium* and *Lactobacillus* while leaving potentially pathogenic populations, including *Clostridium* spp. and *Escherichia coli*, largely unaffected [3,4]. Oligosaccharides are attractive prebiotic candidates because they can be recovered from cheap, abundant biomass, among which agricultural by-products are especially promising [5,6,7]. Fructooligosaccharides, galactooligosaccharides, and inulin are already commercialized [8], and meta-analyses have shown that fructooligosaccharides supplementation can reshape the human gut microbiota [9], including enrichment of beneficial genera such as *Bifidobacterium*, *Lactobacillus*, and *Faecalibacterium* [10,11]. A growing body of work shows that nondigestible oligosaccharides alleviate several human chronic diseases through microbiota regulation [12], that the gut microbiota itself is a central factor in health and disease and interacts with the brain, heart, and metabolic axes [13,14,15], and that polysaccharides from sources such as Moringa leaves or galactooligosaccharides in lactose-intolerant individuals modulate the microbiota and host physiology [16,17,18]. The prebiotic effect of pectin-derived oligosaccharides can also vary with host factors such as age [2,19]. Yet the structural repertoire of plant-derived oligosaccharides remains only partly explored, and some newer candidates show fermentation selectivities that current ingredients do not cover.

Pectin is a structurally complex heteropolysaccharide of the plant primary cell wall. It consists of a homogalacturonan (HG) backbone of α-1,4-linked D-galacturonic acid interspersed with rhamnogalacturonan-I (RG-I) and the highly branched rhamnogalacturonan-II (RG-II) regions, whose side chains are built from up to twelve neutral sugars, including arabinose, galactose, rhamnose, and xylose [20,21]. These neutral side chains, mainly arabinans, galactans, and arabinogalactans attached to the RG-I/RG-II regions, are increasingly regarded as the principal bioactive domains of pectin and as key fermentable substrates for gut microbes [21,22]. Pectic polysaccharides from diverse sources, such as soybean hulls and apple pectin [23,24], share these structural motifs and bioactivities, and plant polysaccharides more broadly are being explored for treating gut dysbiosis and for modulating the gut microbiota and colonic environment [25,26]. Among these sources, apple pectin, recovered from apple pomace (the main by-product of juice processing), is among the most abundant commercial pectins [27,28,29,30]. Native pectin is a fermentable soluble fiber, but its structural complexity and high molecular weight can limit selective microbial utilization. Pectin oligosaccharides (POS) released by partial depolymerization behave differently and have emerged as a new generation of candidate prebiotics [22,31,32] and show additional bioactivities, including improved lipid metabolism [33,34] and effects in food applications such as infant formula [35]; they can be obtained from pectin-rich by-products by chemical, enzymatic, or physical treatments, including enzyme-catalyzed hydrolysis [36,37,38,39,40], microwave-assisted extraction [41], and the preparation of methyl-esterified pectin oligosaccharides with tailored bioactivity [42]. Arabino-oligosaccharides from sugar-beet arabinan and related pectic fractions stimulated bifidobacteria in vitro [43], and structurally distinct pectic fractions were utilized by different microbial groups [44,45].

Crude depolymerization mixtures, however, contain neutral and acidic (galacturonic acid-containing) oligosaccharides together with monosaccharides [22]. Acidic POS can be fermented by a broader and less selective range of gut bacteria, which dilutes the selectivity that defines a prebiotic. Isolating the neutral oligosaccharides, essentially the arabinan, galactan, and arabinogalactan side chains of the RG-I/RG-II regions, is therefore a prerequisite for establishing genuine structure-function relationships [21,22]. Conventional prebiotic testing relies on in vitro batch fermentation with pure or fecal cultures and, eventually, on animal or human trials, all of which are slow, expensive, and hard to standardize. The first committed step of POS utilization is the hydrolysis of glycosidic bonds by microbial glycoside hydrolases (GHs), so the affinity between an oligosaccharide and the GH repertoire of a bacterium is a molecular determinant of its fermentability [46,47,48]. Molecular docking measures this affinity at atomic resolution, and kinetic models of the colonic ecosystem can turn molecular parameters into dynamic predictions of substrate degradation and biomass formation [49,50]. Used together, these two computational layers give a mechanism-based first-pass evaluation before any fermentation experiment is undertaken. Against this background, apple pectin remains understudied relative to its abundance: most existing work addresses its gelling functionality or the crude polysaccharide, whereas the neutral oligosaccharide fraction is still poorly defined structurally, its selective utilization by beneficial versus pathogenic gut bacteria is largely unknown, and a rapid, mechanism-based framework for screening such candidates before biological testing is still lacking.

In this study, we established a streamlined route to obtain structurally defined neutral POS from apple pectin and tested an in silico framework for their prebiotic evaluation. Apple pectin was depolymerized by mild trifluoroacetic acid hydrolysis, and the neutral fraction (P-1) was enriched sequentially by ethanol precipitation, membrane ultrafiltration, and DEAE-Sepharose CL-6B anion-exchange chromatography. The fraction was characterized by monosaccharide composition analysis, HPSEC molecular-weight profiling, and UPLC-HILIC-MS/MS structural elucidation, and its fermentability was predicted by coupling molecular docking against 13 microbial glycosidases with a kinetic simulation of colonic fermentation. By linking the proposed structures of five selected neutral apple pectin oligosaccharides to their predicted utilization by glycosidases from beneficial and pathogenic gut bacteria, this work provides a rapid, mechanism-based screening strategy for pectin-derived prebiotic candidates and a practical route for the valorization of apple pomace.

## 2. Materials and Methods

### 2.1. Materials and Reagents

Commercial food-grade apple pectin was obtained from a local supplier (Fuji cultivar) and used without further purification. Trifluoroacetic acid (TFA), absolute ethanol, sodium chloride, and other analytical-grade reagents were purchased from Sinopharm Chemical Reagent Co., Ltd. (Shanghai, China). DEAE-Sepharose CL-6B was purchased from Solarbio Life Sciences (Beijing, China) and packed into a 30 × 2.6 cm column in our laboratory. Ultrafiltration centrifugal devices (3 kDa cut-off) were obtained from Millipore (Billerica, MA, USA). 1-Phenyl-3-methyl-5-pyrazolone (PMP) and monosaccharide standards were purchased from Sigma-Aldrich (Shanghai, China). MS-grade acetonitrile was obtained from Merck & Co. (Kenilworth, NJ, USA). A structurally defined synthetic pectin was deliberately not used: this work targets a food-grade industrial feedstock, and the same commercial material characterized in our previous study [1] was chosen so that the results remain directly comparable and transferable to apple pomace valorization. The pectin was extracted from Fuji apples harvested in the picking season (around October), and the fruit supplied was of food-grade quality.

### 2.2. Preparation of Neutral Pectin Oligosaccharides

**Figure 1:**
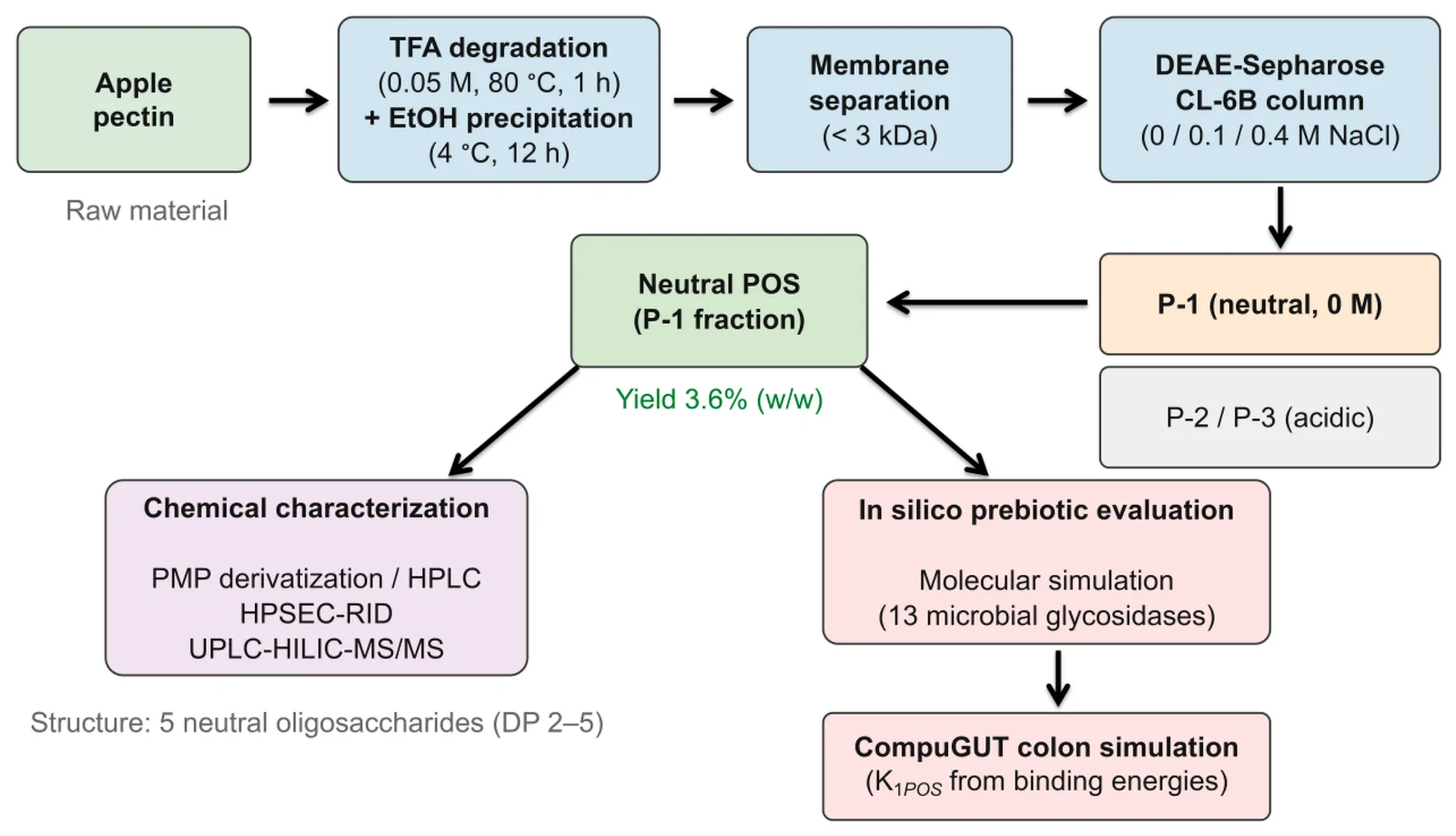
Integrated workflow for the preparation, purification, structural characterization, and in silico prebiotic evaluation of neutral pectin oligosaccharides (POS) from apple pectin.

The overall workflow is shown in Figure 1. An apple pectin solution (1 g/L) was hydrolyzed with 0.05 M TFA at 80 °C for 1 h. These mild conditions preferentially depolymerize the neutral-sugar side chains while suppressing intramolecular β-elimination of the galacturonan backbone, which is negligible below pH 4 [20]. To the hydrolysate (400 mL), absolute ethanol (600 mL) was added, and the mixture was kept at 4 °C for 12 h to precipitate high-molecular-weight pectic material. After centrifugation, the supernatant was concentrated under reduced pressure at 40 °C, filtered through a 0.45 μm aqueous membrane, and fractionated in a 3 kDa ultrafiltration centrifugal device [3]. The permeate (<3 kDa) was concentrated at 40 °C and freeze-dried.

The resulting oligosaccharide mixture (50 mg) was loaded onto a laboratory-packed DEAE-Sepharose CL-6B anion-exchange column (30 × 2.6 cm) pre-equilibrated with ultrapure water [51]. Stepwise elution was performed with water (0 M NaCl), 0.1 M NaCl, and 0.4 M NaCl at a flow rate of 1 mL/min, with elution times of 720, 810, and 450 min, respectively; fractions of 9 mL were collected. The total sugar content of each tube was determined, and the elution curve was constructed by plotting the sugar content against the elution volume. The unbound fraction eluted with water (P-1) was pooled, concentrated, and freeze-dried for subsequent analyses.

### 2.3. Characterization of the Neutral POS

#### 2.3.1. Total Sugar and Monosaccharide Composition

Total sugar content was measured by the microplate phenol-sulfuric acid assay [43]. Monosaccharide composition was determined by pre-column PMP derivatization [5]. Briefly, the sample (4 mg) was hydrolyzed with 4 M TFA (1 mL) at 110 °C for 4 h under nitrogen, and TFA was removed by repeated methanol evaporation. The hydrolysate was dissolved in water (100 μL), alkalinized with 0.6 M NaOH (100 μL), and derivatized with 0.3 M PMP in methanol (50 μL of sample solution plus 50 μL of reagent) at 70 °C for 100 min. After neutralization with 0.3 M HCl (200 μL) and extraction with chloroform (three times), the aqueous layer was filtered (0.45 μm) and analyzed on an Agilent 1260 HPLC system equipped with an HC-C18 column. Gradient elution was performed with 15% acetonitrile/85% phosphate buffer (pH 6.7) (eluent A) and 40% acetonitrile/60% phosphate buffer (eluent B) at 1.0 mL/min, with UV detection at 254 nm and the column temperature maintained at 25 °C.

#### 2.3.2. Molecular-Weight Distribution

The molecular-weight distribution was assessed by high-performance size-exclusion chromatography (HPSEC) [22]. Samples (5 mg/mL in ultrapure water, 0.22 μm filtered) were analyzed on an Agilent 1260 system fitted with a TSKgel GMPWXL column and a refractive-index detector at 35 °C. Isocratic elution with ultrapure water was performed at 1.0 mL/min for 55 min. A 1 kDa dextran standard was used as the molecular-weight reference.

#### 2.3.3. Structural Elucidation by UPLC-HILIC-MS/MS

Oligosaccharide structures were analyzed by ultra-performance liquid chromatography coupled to tandem mass spectrometry (UPLC-HILIC-MS/MS) [52]. Chromatographic separation was performed on a Waters ACQUITY UPLC system equipped with an ACQUITY UPLC BEH Amide column (2.1 × 150 mm, 1.7 μm) in combination with a Vanguard precolumn (2.1 × 5 mm, 1.7 μm; Waters Corp., Milford, MA, USA) maintained at 35 °C. The mobile phase consisted of acetonitrile containing 0.1% formic acid (eluent A) and water containing 10 mM ammonium acetate and 0.1% formic acid (eluent B), delivered at 0.3 mL/min with the following gradient: 0–1 min, isocratic 80% A; 1–25 min, linear from 80% to 50% A; 25–30 min, linear from 50% to 80% A; 30–35 min, isocratic 80% A; the injection volume was 1 μL. Mass spectra were recorded on a Waters Q-Tof mass spectrometer (Waters Corp., Milford, MA, USA) operated in positive electrospray ionization (ESI+) mode with capillary voltage 3.0 kV, cone voltage 40 V, source temperature 100 °C, desolvation temperature 400 °C, cone gas flow 50 L/h, and desolvation gas flow 800 L/h. The full-scan range was *m*/*z* 100–2000, and MS/MS spectra were acquired over *m*/*z* 100–1000 in MS/MS (product-ion) scan mode with collision-induced dissociation [52]. Structures were assigned from the precursor ions and the diagnostic glycosidic and cross-ring fragments of the MS/MS spectra [52,53].

### 2.4. In Silico Evaluation of Prebiotic Potential

#### 2.4.1. Molecular Simulation of POS with Microbial Glycosidases

Three-dimensional structures of the oligosaccharides were generated with the GLYCAM-Web Carbohydrate Builder at https://glycam.org/cb/ (accessed on 10 October 2024) and saved in Mol2 format. These structures are the proposed assignments derived from the MS/MS evidence (Section 3.3) and were treated as tentative throughout the computational analysis. All five components were docked against each of the 13 glycosidases. The galactan A carries only β-D-Galp linkages and the arabinan B only α-L-Araf linkages, so the two components are recognized by mutually exclusive enzyme classes; their binding energies are therefore reported in a single A/B column (Section 3.4.1), in which each row gives the value of the component matched to that enzyme’s class—the arabinan B for the arabinosidase class and the galactan A for the β-galactosidase class (Section 3.4.1). Galactose- and arabinose-active glycoside hydrolases of the human gut microbiota were selected from the CAZy database; the corresponding protein sequences were retrieved from UniProt, and three-dimensional models were predicted using the deep-learning models RoseTTAFold and AlphaFold2. Thirteen enzymes were retained, covering beneficial bacteria (healthy group, H: *Bacteroides ovatus*, *Butyrivibrio fibrisolvens*, *Bifidobacterium bifidum*, *B. adolescentis*, *B. longum* subsp. *infantis*, *Lactobacillus helveticus*, and *L. delbrueckii* subsp. *bulgaricus*), a commensal reference (other group, O: *Bacillus subtilis*), and pathogens (pathogenic group, P: *Escherichia coli* O157:H7 and *Klebsiella pneumoniae*). Docking calculations were conducted using Molecular Operating Environment (MOE) and AutoDock 4.2.6 software packages [54]. Docking used the induced-fit protocol with Triangle Matcher placement, London dG rescoring, force-field refinement, and GBVI/WSA dG final rescoring (3000 retained poses). The binding energy (kcal/mol) of the best pose was recorded for each oligosaccharide–enzyme pair; more negative values indicate stronger binding. The predicted protein models were retained only when the per-residue confidence scores of the deep-learning predictions indicated reliably folded catalytic domains, and the carbohydrate-binding clefts identified from the CAZy/UniProt annotations and from homologous crystal structures were used as the docking search space. The induced-fit protocol, in which 3000 poses are generated and reranked by GBVI/WSA dG rescoring, provides an internal consistency control, and independent repeated docking runs were performed to confirm the reproducibility of the reported scores.

#### 2.4.2. Colon Fermentation Simulation

The dynamic fate of the neutral POS in the colonic environment was simulated with an in silico intestinal fermentation platform [44,49]. The docking-derived binding energies were converted into compound-specific half-saturation constants (K_1*POS*_, g/gXsu) through the Arrhenius and Monod relationships and were used to replace the default K1 value of standard fiber in the model. The temporal evolution of substrate hydrolysis and of total biomass, defined as the sum of microbial mass, released oligosaccharides, short-chain fatty acids, and gas, was monitored in the lumen and in the mucosal layer [50,54]. The simulation was run for 28 days; the first 48 h (2 days) of the trajectories, covering the window within which substrate hydrolysis was essentially complete, are presented in the main text, and the complete 28-day simulations are provided as animated GIF files in the Supplementary Material. Briefly, the docking scores were first converted into binding free energies (ΔG = RT ln Kd) and then into first-order hydrolysis rate constants through the Arrhenius relationship, assuming a common pre-exponential factor across this homologous glycosidase series; the resulting constants were scaled to Monod half-saturation constants (K_1*POS*_) as described previously [49]. For each component, the resulting per-enzyme constants were then averaged with weights reflecting the enzyme classes able to hydrolyze its linkages: component A, a β-D-galactan, is a substrate only for the β-galactosidase class, whereas component B, a linear (1→4)-α-L-arabinan, is a substrate only for the arabinosidase class, so the K_1*POS*_ values of A and B are averaged over different, mutually exclusive enzyme sets and therefore differ (5.39 × 10^−3^ and 1.59 × 10^−3^ g/gXsu, respectively). All other model parameters were kept at their default, experimentally calibrated values, and the absolute fermentation profiles should be read as qualitative rankings, because the conversion has not yet been calibrated against experimental kinetic datasets.

### 2.5. Statistical Analysis

All chemical and chromatographic analyses (total sugar, monosaccharide composition, HPSEC, and UPLC-HILIC-MS/MS) were performed in triplicate (*n* = 3), and the results are reported as mean values. Molecular docking scores and colon fermentation simulation outputs are deterministic results of the respective computational models.

## 3. Results and Discussion

### 3.1. Preparation and Purification of the Neutral POS

Apple pectin consists of a long galacturonan backbone, and its RG-II region carries side chains built from up to twelve different neutral sugars [20,21]. The preparation route (Figure 1) was designed to cleave these neutral-sugar-rich regions while keeping the pH below 4, where intramolecular β-elimination of the pectic backbone is negligible [20]. Mild TFA degradation, ethanol precipitation, membrane ultrafiltration, and anion-exchange chromatography then enriched the target molecules sequentially by size and by charge.

**Figure 2:**
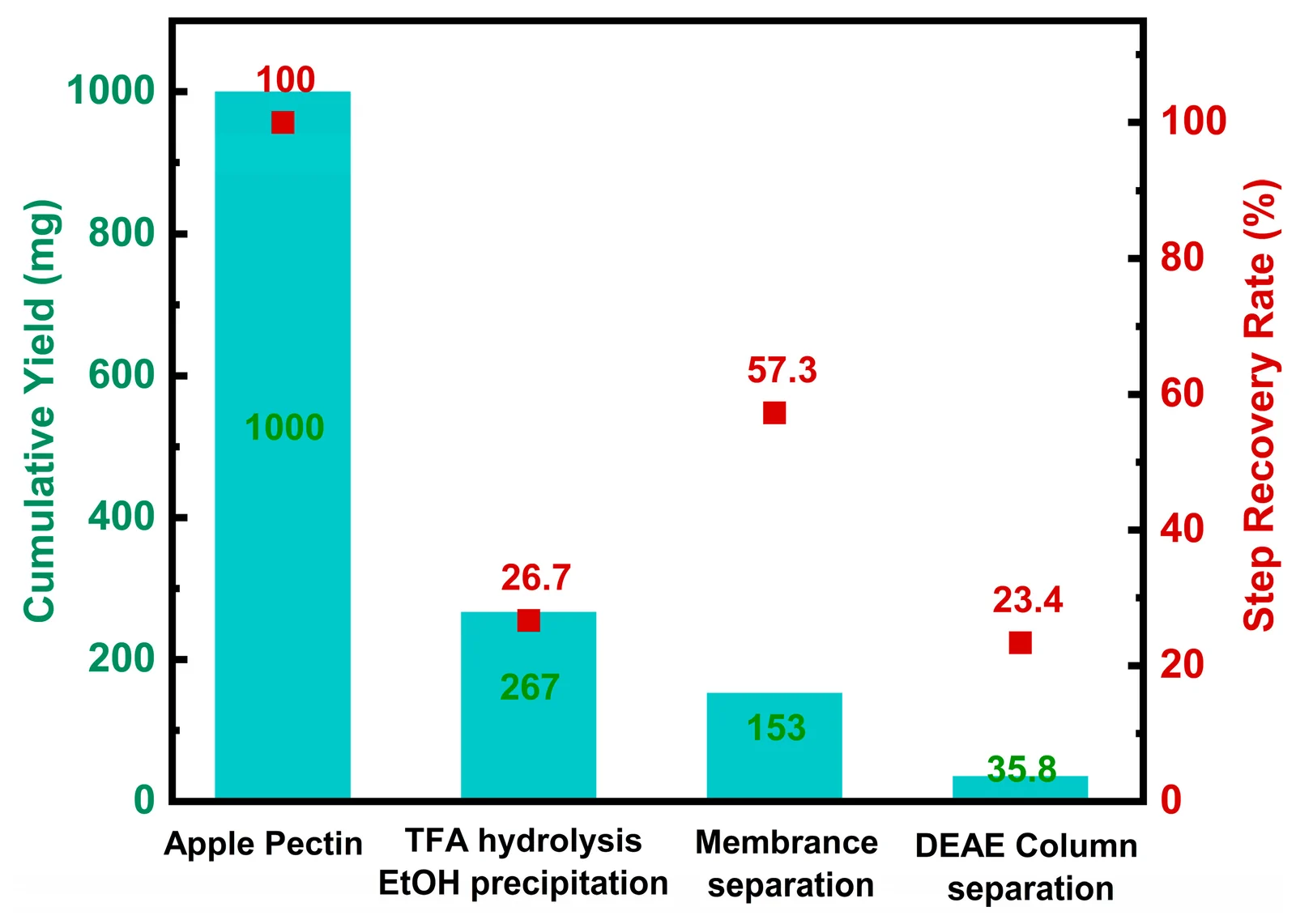
Cumulative yield (bars) and step recovery rate (squares) of the neutral POS along the multi-step purification process.

The mass balance of the process is summarized in Figure 2. Starting from 1000 mg of apple pectin, the sequence of hydrolysis, ethanol precipitation, membrane separation, and anion-exchange chromatography afforded 35.8 mg of the neutral P-1 fraction, corresponding to an overall yield of about 3.6% on a pectin basis; the intermediate masses and step recoveries are given in the figure and are not repeated here. The yield is modest but consistent with the abundance of the neutral RG-II side chains relative to the galacturonan backbone, and the process should scale well given the low cost of the raw material. It should also be noted that residual TFA has to be removed exhaustively before any food-grade application, a downstream burden that enzymatic routes do not carry.

**Figure 3:**
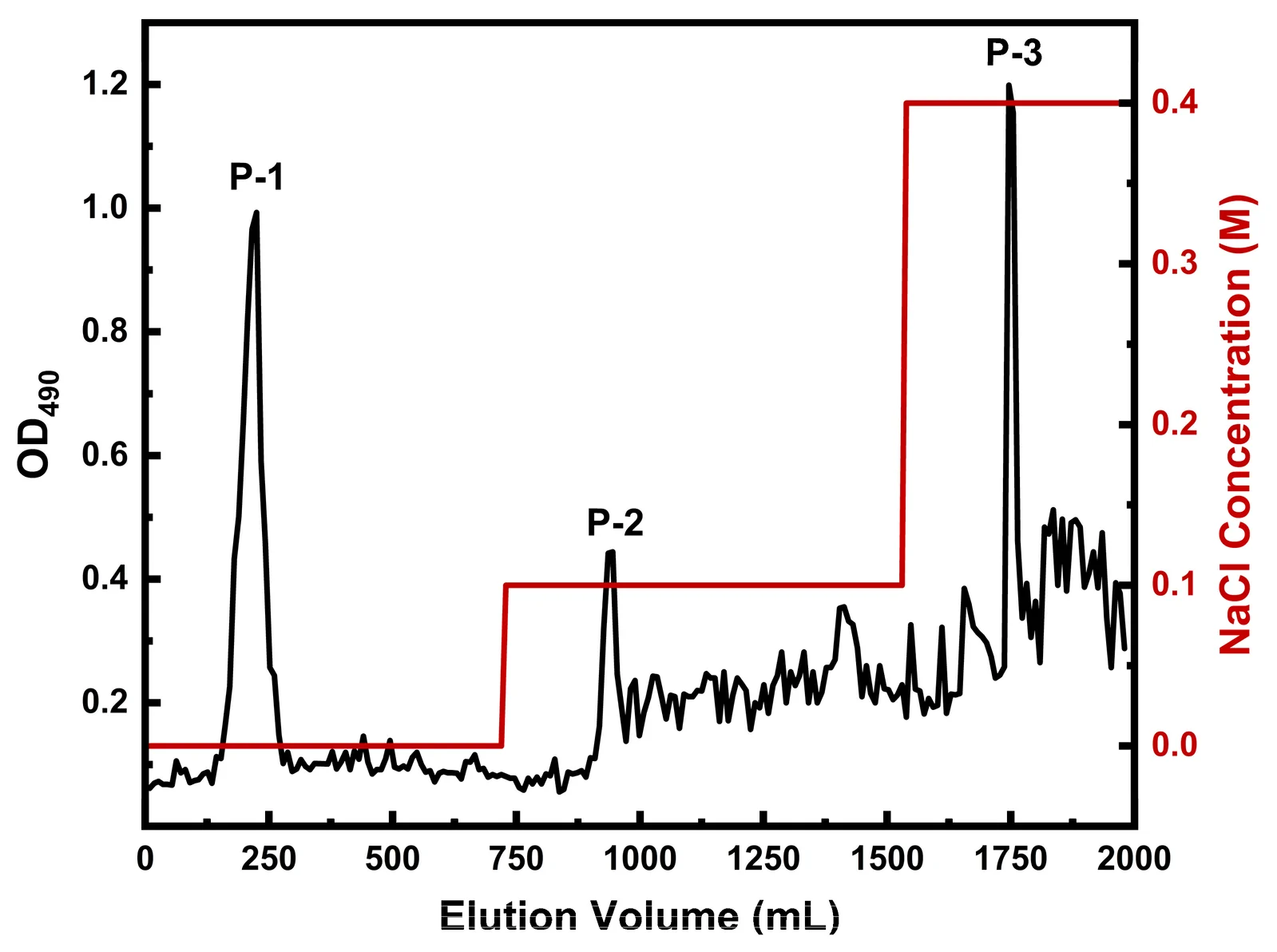
Elution profile of the < 3 kDa POS fraction on the DEAE-Sepharose CL-6B column. P-1 (eluted with water, 0 M NaCl): neutral POS; P-2 and P-3 (0.1 and 0.4 M NaCl): acidic POS fractions. The red trace indicates the NaCl step gradient.

The elution profile of the <3 kDa fraction on the DEAE-Sepharose CL-6B column (Figure 3) resolved three main populations [51]. P-1 came out with water (0 M NaCl) and, by the anion-exchange mechanism, corresponds to oligosaccharides that carry no negative charge and are not retained by the resin, i.e., the neutral POS. P-2 and P-3, eluted with 0.1 M and 0.4 M NaCl, contain acidic oligosaccharides with increasing galacturonic acid content. Weighing the neutral character of P-1 against the recovery cost, P-1 was selected for all subsequent characterization and evaluation.

### 3.2. Monosaccharide Composition and Molecular-Weight Distribution

The monosaccharide compositions of the raw pectin and of the P-1 fraction are compared in Table 1. The raw apple pectin was dominated by galacturonic acid (63.67 mol%), consistent with its galacturonan backbone. Ethanol precipitation already brought the GalA content down to 4.35 mol%, and the P-1 fraction contained only 0.16 mol% GalA, together with 41.70 mol% galactose, 31.37 mol% glucose, 19.16 mol% arabinose, and minor proportions of xylose (6.36 mol%), mannose (0.70 mol%), and rhamnose (0.55 mol%). Size-based ultrafiltration followed by charge-based anion exchange thus stripped the acidic galacturonan material and left an essentially neutral oligosaccharide pool. The enrichment in galactose and arabinose is the compositional signature of the arabinogalactan and arabinan side chains of the pectic RG regions [21,22]. The substantial glucose proportion (31.37 mol%) warrants clarification. The PMP-HPLC analysis reports every carbohydrate species released by TFA hydrolysis of the fraction, including residual free monosaccharides and co-extracted glucan material (e.g., starch- or cellulose-derived fragments) that accompany pectin in commercial preparations, so Table 1 describes the bulk composition of P-1 rather than the residue composition of individual oligosaccharides. Consistent with this, none of the five MS/MS-resolved oligosaccharides contains glucose. These five components were selected for the in silico evaluation because they are the structurally assignable constituents of the fraction. The full chromatographic profile is more complex than these five species (Figure S1), which therefore constitute a selected, tentatively assigned subset of P-1 rather than demonstrated dominant or representative components; their individual relative abundances could not be quantified from the available data, and the in silico results below are accordingly interpreted for these five selected compounds only.

**Figure 4:**
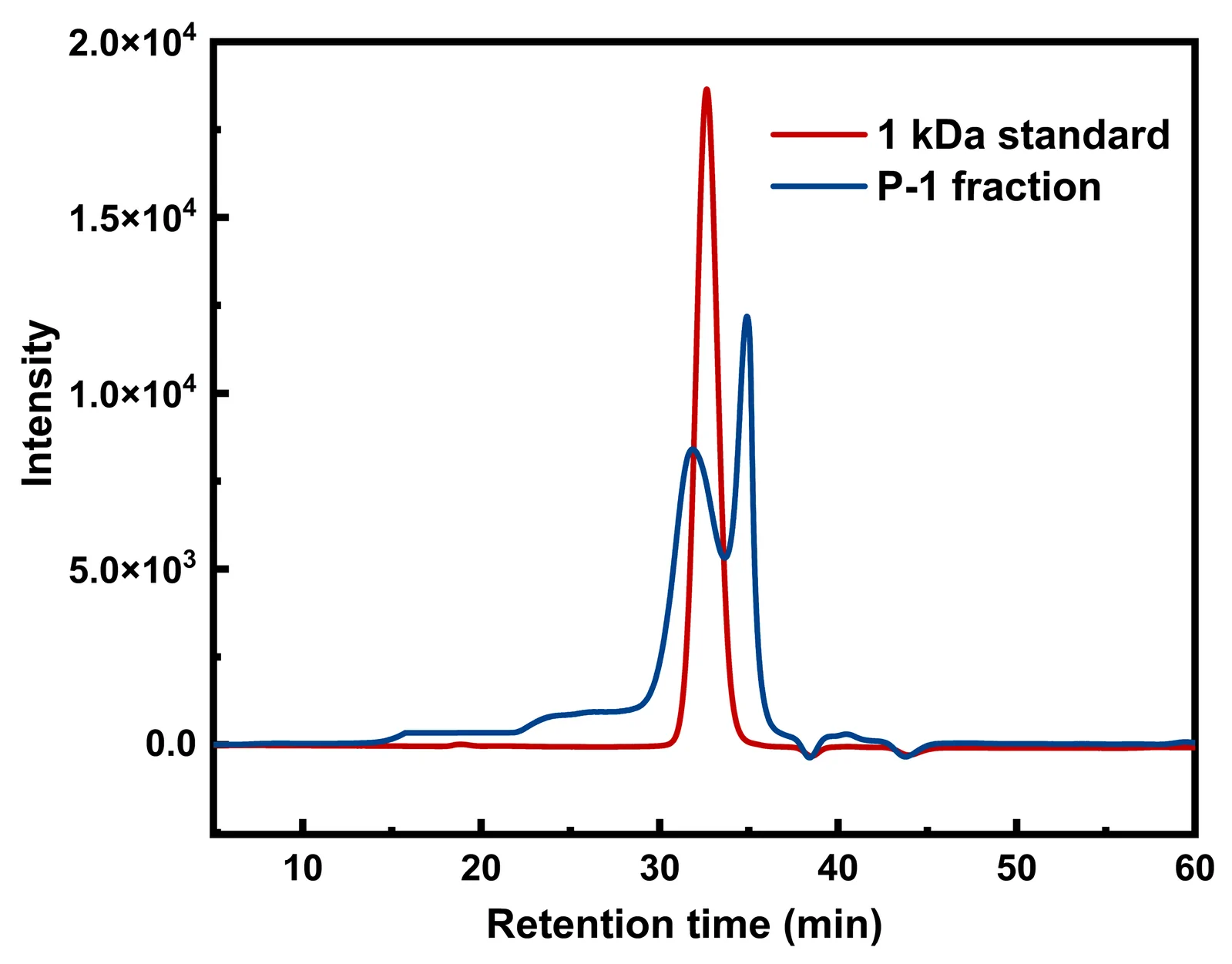
HPSEC-RID chromatograms of the P-1 fraction (blue) and of the 1 kDa dextran standard (red).

HPSEC analysis (Figure 4) showed that P-1 co-eluted close to the 1 kDa dextran standard, giving a molecular-weight distribution centered near 1 kDa and corresponding to degrees of polymerization (DP) of roughly 2–5. The two partially resolved P-1 peaks flanking the standard point to at least two oligosaccharide subpopulations of slightly different hydrodynamic volume, in line with the structural heterogeneity later resolved by mass spectrometry (Section 3.3). Oligosaccharides in this DP window are readily accessible to microbial glycosidases while keeping fermentation selectivity [43,45].

### 3.3. Structural Elucidation of the Neutral POS by UPLC-HILIC-MS/MS

Five neutral oligosaccharides were selected from the chromatographic profile and tentatively assigned by UPLC-HILIC-MS/MS (Table 2). All are composed exclusively of β-D-galactopyranose (β-D-Galp) and α-L-arabinofuranose (α-L-Araf) residues connected through (1→4) and (1→6) glycosidic linkages, and all are devoid of galacturonic acid, which confirms at the molecular level the neutrality of the P-1 pool seen in the compositional analysis.

Assigning the fragmentation patterns was straightforward. The disaccharide A (*m*/*z* 362.25) gave the expected glycosidic fragments of a galactobiose, and its (1→4) linkage followed from the relative intensities of the cross-ring cleavage ions. The arabinogalactans C and D displayed the diagnostic 162 Da (hexose) and 132 Da (pentose) losses of terminal galactose and arabinose residues, so the β-D-Galp and α-L-Araf sequence could be reconstructed residue by residue; the mixed (1→4)/(1→6) connectivities were assigned from the cross-ring fragments. Component B (*m*/*z* 453.35) showed the consecutive loss of two 132 Da pentose units and was identified as a linear (1→4)-linked arabinotriose. Component C (*m*/*z* 475.33), a DP3 arabinogalactan, and component E (*m*/*z* 701.49), the DP5 arabinogalactan, displayed the characteristic hexose and pentose losses expected of mixed galactose/arabinose chains. These molecules correspond to the arabinan and type-II arabinogalactan side chains characteristic of the RG-I/RG-II pectic region [20,21]. Their structures agree fully with the monosaccharide composition, rich in galactose and arabinose and essentially free of galacturonic acid, and with the DP 2–5 window estimated by HPSEC. It should be stressed that the monosaccharide identities, the anomeric configurations (β-D-Galp and α-L-Araf), and the (1→4) and (1→6) linkage positions are proposed assignments that combine the MS/MS fragmentation evidence with established structural knowledge of pectic arabinan and arabinogalactan side chains [20,21]; MS/MS data alone cannot unambiguously define these features. The observed *m*/*z* values are therefore reported together with their proposed adduct assignments, and the complete chromatograms and raw MS/MS spectra are provided as Supplementary Material (Figures S1–S3) for independent inspection. The heterogeneity of the proposed ionic forms ([M+K–H_2_O]^+^, [M+K]^+^, [M+H]^+^, [M+H–H_2_O]^+^, and [M+NH_4_–3H_2_O]^+^) reflects the ionization behaviour of neutral oligosaccharides in positive ESI: lacking basic sites, these molecules ionize mainly through proton, alkali-metal, or ammonium adduction, the potassium adducts plausibly arising from residual K^+^ associated with the pectin matrix, while the water-loss species are consistent with the ready dehydration of labile glycosidic structures under the source conditions. For each component, the adopted adduct assignment was the one giving the smallest deviation between the measured and the theoretical mass (Table 2).

### 3.4. In Silico Evaluation of the Prebiotic Potential

#### 3.4.1. Molecular Simulation with Microbial Glycosidases

The first committed step in the microbial utilization of an oligosaccharide is its hydrolysis by glycoside hydrolases, so the affinity of a POS for the glycosidase repertoire of a bacterium is a molecular proxy of its fermentability [46,47]. Table 3 compiles the binding energies of the proposed structures of the five neutral POS with 13 glycosidases from healthy gut microbiota (H), other species (O), and pathogenic microorganisms (P). Docking covered arabinosidase-class enzymes (class A, active on α-L-Araf linkages) and β-galactosidase-class enzymes (class B, active on β-D-Galp linkages). As stated in Section 2.4.1, all five components were docked against each enzyme, and the A and B labels in Table 3 denote the two enzyme groups—the docking targets: class A comprises P49943, P45982, and P42293, and class B comprises A7LXS9, D4QFE7, A1A399, Q93GI5, O07013, O07012, Q7WTB4, Q1G9Z4, Q8X685, and A7KGA5. Because the galactan A and the arabinan B are recognized by mutually exclusive enzyme classes, their binding energies are reported in a single A/B column in which each row gives the value of the component matched to that enzyme’s class (see the Table 3 note).

Several consistent features emerge from the docking matrix. Components A and B bound more weakly than component C and the larger arabinogalactans D and E across the enzyme panel, which suggests that the larger, mixed-sugar scaffolds complement the glycosidase active sites better, both sterically and electrostatically. Because the galactan A and the arabinan B were each evaluated against the enzyme class matching their linkages (Table 3), the matrix compares the two only within their respective classes, and no direct galactan-versus-arabinan comparison is drawn. Component E, the DP5 arabinogalactan, gave the strongest overall affinities and reached −10.3 kcal/mol with the β-galactosidase of *B. longum* subsp. *infantis* (Q93GI5). The DP4 arabinogalactan (component D) was the next strongest with the β-galactosidase of *B. ovatus* (A7LXS9) at −8.9 kcal/mol. The pathogen enzymes behaved differently: binding of component E to Q8X685 (*E. coli* O157:H7) and A7KGA5 (*K. pneumoniae*) reached only −7.4 and −6.1 kcal/mol, the two weakest values recorded for this compound [47,48].

**Figure 5:**
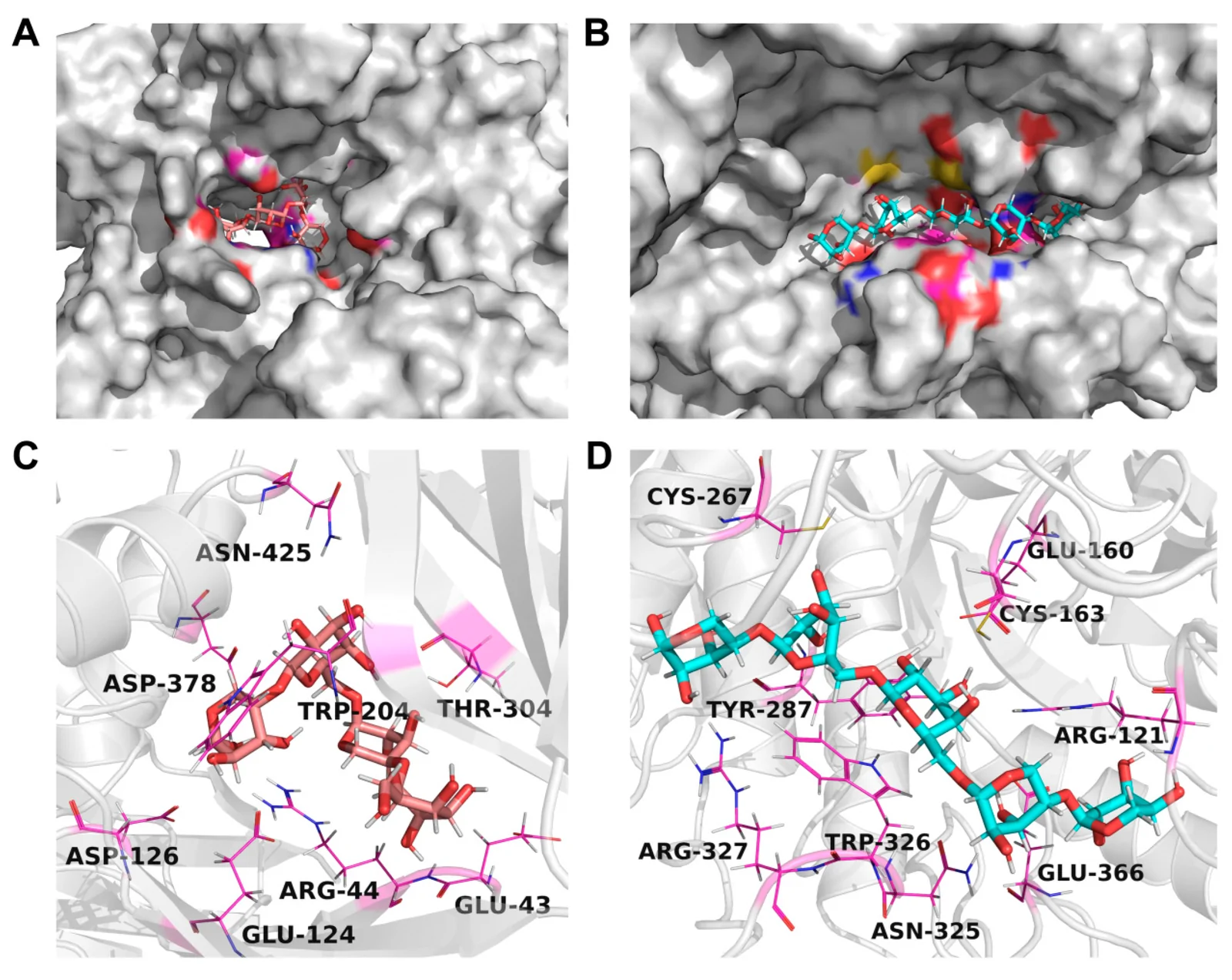
Molecular simulation poses of the two strongest-binding oligosaccharide–enzyme complexes. (**A**,**C**) DP4 arabinogalactan (component D) bound to the β-galactosidase of *Bacteroides ovatus* (A7LXS9; binding energy −8.9 kcal/mol): surface view and residue-interaction view. (**B**,**D**) DP5 arabinogalactan (component E) bound to the β-galactosidase of *B. longum* subsp. *infantis* (Q93GI5; −10.3 kcal/mol). The protein is shown as a gray solvent-accessible surface (**A**,**B**) or cartoon (**C**,**D**), the oligosaccharide ligands are shown as sticks (red for component D, cyan for component E), and the interacting residues are shown as magenta sticks with black labels; oxygen and nitrogen atoms are colored red and blue, respectively.

The two strongest-binding complexes (those with the most negative docking energies) illustrate the structural origin of these affinities (Figure 5). In the complex of the DP4 arabinogalactan (component D) with the *B. ovatus* β-galactosidase A7LXS9 (Figure 5A,C), the ligand lies deep in the catalytic pocket. Its hydroxyl groups contact ARG44, GLU43, GLU124, ASP126, TRP204, THR304, ASP378, and ASN425, and the two acidic residues ASP126 and ASP378 lie close to the glycosidic oxygen, in the arrangement expected for the catalytic machinery of a retaining β-galactosidase. In the complex of the longer DP5 chain (component E) with the *B. longum* subsp. *infantis* β-galactosidase Q93GI5 (Figure 5B,D), the oligosaccharide binds along an extended surface groove, contacting CYS163, ARG121, ASN325, GLU366, TRP326, TYR287, ARG327, CYS267, and GLU160. The alternating (1→4) and (1→6) linkages let the chain follow the curvature of this groove, which probably explains why affinity increases with chain length within the arabinogalactan series. In both complexes the sugar hydroxyls face polar and charged residues while aromatic side chains (TRP, TYR) pack against the pyranose rings, a recurrent geometry of carbohydrate-recognition sites. These binding poses are consistent with the reading of Table 3: the proposed structures of the five selected POS engage the glycosidases of beneficial bacteria productively, and the engagement weakens for the pathogen enzymes [47,48].

#### 3.4.2. Colon Fermentation Dynamics

To translate these molecular affinities into predictions of colonic fermentability, the binding energies were converted into compound-specific half-saturation constants (K_1*POS*_) through the Arrhenius and Monod relationships and introduced into the colon fermentation model [49]. The constants (Table 4) spanned more than an order of magnitude, from 5.39 × 10^−3^ g/gXsu for component A to 3.97 × 10^−4^ g/gXsu for component E. Components A and B received different constants (5.39 × 10^−3^ and 1.59 × 10^−3^ g/gXsu) because their per-enzyme constants are averaged over different, mutually exclusive enzyme classes: the galactan A over the β-galactosidase class, and the linear arabinan B over the arabinosidase class (Section 2.4.2). A lower K_1*POS*_ means a higher effective affinity of the colonic enzymatic machinery for the substrate, so component E, which also had the strongest docking affinity, is predicted to be the most readily fermentable of the five oligosaccharides.

**Figure 6:**
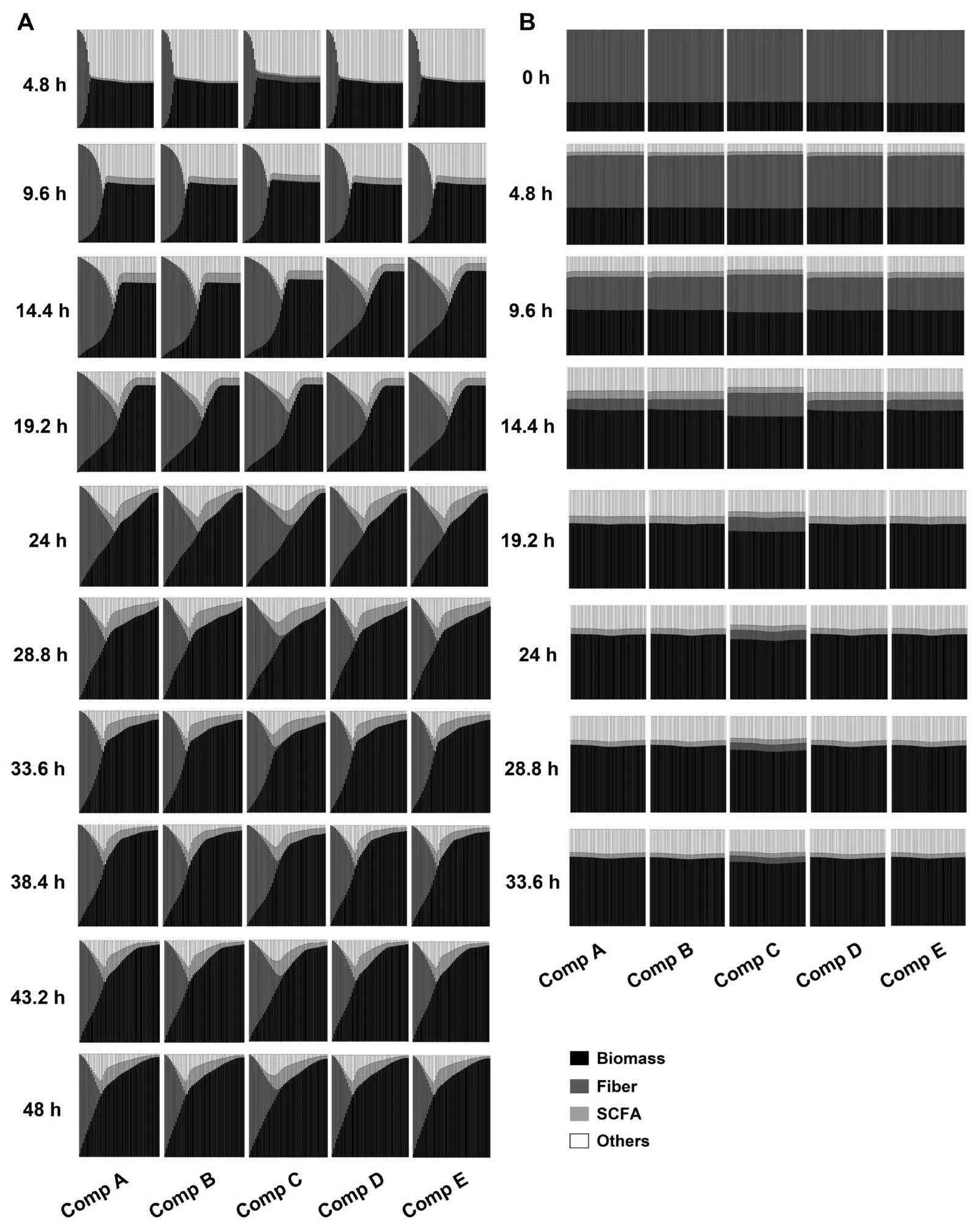
CompuGUT simulation of the colonic fermentation of the five neutral POS (components A–E) over the first 48 h (2 days) of the 28-day simulation. (**A**) Intestinal lumen: temporal evolution of substrate hydrolysis. (**B**) Mucosal layer: evolution of the total biomass (microbial mass + released oligosaccharides + short-chain fatty acids + gas). The static figure thus represents only the initial 2 days of the run; the complete time-resolved simulations of each component in both compartments are provided as animated GIF files (28 days), with static end-frame previews assembled in Figure S4 of the Supplementary Material. Each animation shows the temporal evolution of the simulation in the corresponding compartment (biomass, fiber, SCFA, and other components as indicated in the legend).

The simulated fermentation dynamics over 48 h are presented in Figure 6. These static panels show only the first 48 h (2 days) of the 28-day simulation (see Section 2.4.2). In the colonic lumen (Figure 6A), each of the five POS was progressively hydrolyzed: the hydrolyzed fraction (dark area) expanded continuously from 4.8 h onward and hydrolysis was essentially complete within 48 h for all components. The five selected oligosaccharides are therefore predicted to be readily hydrolyzable in the intestinal environment, a trend consistent with that reported for other readily fermentable pectic oligosaccharides [22]. It should be noted that the simulation is parameterized with the docking-derived energies, so the agreement between the docking ranking and the simulated hydrolysis behaviour is expected by construction and reflects the internal consistency of the pipeline rather than independent validation of one approach by the other. At comparable degrees of polymerization, the neutral oligosaccharides were predicted to hydrolyze faster than their acidic counterparts, which further supports the neutral-sugar purification strategy [5]. In the mucosal layer (Figure 6B), all five components promoted the total biomass, defined as the sum of microbial mass, released oligosaccharides, short-chain fatty acids, and gas, throughout the simulation. The fermentation products of these five selected oligosaccharides are thus predicted to support microbial growth and metabolism at the mucosal surface, where most host–microbiota interactions take place [50,55,56]. Short-chain fatty acids released by this fermentation, and the organisms that produce them, are increasingly recognized as relevant to intestinal and systemic health [57,58,59], and the RG-I domain is known to shape the in vitro fermentation of pectic polysaccharides [60]; related in vitro fermentations of galacturonan oligosaccharides likewise show that pectic fragments are rapidly and selectively metabolized [61].

The docking and simulation results point the same way, as expected for two coupled layers in which the simulation is parameterized by the docking output; their agreement indicates internal consistency rather than independent validation. Within this frame, the five selected oligosaccharides from apple pectin bind preferentially to the glycosidases of beneficial gut bacteria, are predicted to hydrolyze rapidly in the colonic lumen, and promote mucosal biomass formation in the model, while remaining poorly recognized by the enzymes of *E. coli* O157:H7 and *K. pneumoniae*. Docking to individual glycosidases does not by itself demonstrate microbial utilization, growth modulation, or prebiotic activity, and a comparison restricted to the enzymes of two pathogenic species cannot establish selective utilization by the gut microbiota as a whole. The DP5 arabinogalactan (component E) performed best on all three criteria. Mixed galactose/arabinose composition, higher DP, and mixed (1→4)/(1→6) linkages therefore appear to favor the predicted selective utilization of these five compounds, in agreement with the selectivity reported for pectic oligosaccharides of related structures [22,43,44]. The present evaluation is computational, and in vitro fecal fermentation and ultimately in vivo studies will be needed to confirm these predictions. Even so, structural elucidation combined with docking against a curated glycosidase panel and kinetic colon simulation gives a fast, mechanism-based screen that can rank candidates before any laborious biological testing [44,49].

## 4. Conclusions

Starting from commercial apple pectin, we prepared a neutral pectin oligosaccharide fraction (P-1) through mild TFA degradation, ethanol precipitation, membrane ultrafiltration, and DEAE-Sepharose CL-6B chromatography, in 3.6% overall yield and with the galacturonic acid content reduced from 63.67 to 0.16 mol%. UPLC-HILIC-MS/MS resolved five selected neutral oligosaccharides (DP 2–5) built from β-D-Galp and α-L-Araf through (1→4) and (1→6) linkages, assignable to the arabinan and arabinogalactan side chains of the RG-I/RG-II pectic region. All five compounds bound more strongly to glycosidases from beneficial gut bacteria than to those from the pathogens *E. coli* O157:H7 and *K. pneumoniae* within the present enzyme panel. The DP5 arabinogalactan showed the highest overall affinity (−10.3 kcal/mol with the *B. longum* subsp. *infantis* β-galactosidase) and the lowest half-saturation constant (3.97 × 10^−4^ g/gXsu), and colon simulation predicted rapid luminal hydrolysis and a sustained increase of mucosal biomass. These five selected oligosaccharides are thus predicted in silico to be fermentable and to engage preferentially the glycosidases of beneficial bacteria. Because the predictions rest on five proposed structures whose quantitative contribution to P-1 is unknown, they apply to these compounds only and cannot be generalized to the fraction as a whole; they define a prioritized candidate list for subsequent experimental evaluation rather than demonstrated or predicted prebiotic activity of P-1. Follow-up work will test these predictions in in vitro fecal fermentations and in vivo, scale up the purification, and apply the same evaluation framework to other pectin-rich agro-industrial by-products.

## Figures and Tables

**Table 1:** Monosaccharide composition (mol%) of the raw apple pectin and of the purified neutral POS fraction (P-1). Values are means of triplicate determinations (*n* = 3).

Sample	Monosaccharides (mol%)						
	Mannose	Rhamnose	Galacturonic Acid	Galactose	Glucose	Arabinose	Xylose
Apple pectin	4.23	6.01	63.67	8.31	6.91	7.93	2.94
P-1	0.70	0.55	0.16	41.70	31.37	19.16	6.36

**Table 2:** UPLC-HILIC-MS/MS spectra and proposed structures of the five neutral oligosaccharides identified in the P-1 fraction.

Comp	t_R_/min	*m*/*z*	Ionic Form	Measured Mass (Mass Error)	Proposed Structure
A	3.83	362.25	[M+K-H_2_O]^+^	341.25 (+0.87)	β-D-Galp-(1→4)-β-D-Galp
B	11.44	453.35	[M+K]^+^	414.35 (−0.21)	α-L-Araf-(1→4)-α-L-Araf-(1→4)-α-L-Araf
C	5.40	475.33	[M+H]^+^	474.33 (−0.17)	β-D-Galp-(1→6)-β-D-Galp-(1→4)-α-L-Araf
D	7.02	588.41	[M+H-H_2_O]^+^	605.41 (+0.79)	β-D-Galp-(1→4)-α-L-Araf-(1→6)-β-D-Galp-(1→4)-α-L-Araf
E	8.56	701.49	[M+NH_4_-3H_2_O]^+^	737.49 (+0.75)	α-L-Araf-(1→4)-α-L-Araf-(1→6)-β-D-Galp-(1→6)-β-D-Galp-(1→4)-α-L-Araf

**Table 3:** Binding energies (kcal/mol) of the neutral POS with glycosidases from healthy gut microbiota (H), other species (O), and pathogenic microorganisms (P).

UniProt	Enzyme	Species (Type)	Binding Energy (kcal/mol)				
			A/B *		C	D	E
P49943	Arabinosidase	*Bacteroides ovatus* (H)	**A**	−6.1	−6.4	−7.4	−7.7
P45982	Arabinosidase	*Butyrivibrio fibrisolvens* (H)		−6.4	−6.7	−6.8	−8.3
P42293	Arabinanase	*Bacillus subtilis* (O)		−6.3	−6.7	−7.9	−7.8
A7LXS9	β-galactosidase	*Bacteroides ovatus* (H)	**B**	−7.1	−7.8	−8.9	−8.1
D4QFE7	β-galactosidase	*Bifidobacterium bifidum* (H)		−5.8	−7.1	−7.6	−8.5
A1A399	β-galactosidase	*Bifidobacterium adolescentis* (H)		−6.0	−6.3	−6.9	−8.0
Q93GI5	β-galactosidase	*Bifidobacterium longum* subsp. *Infantis* (H)		−5.7	−6.4	−7.5	−10.3
O07013	Arabinogalactan galactanase	*Bacillus subtilis* (O)		−5.7	−6.7	−7.2	−8.6
O07012	β-galactosidase	*Bacillus subtilis* (O)		−5.4	−6.2	−6.7	−7.6
Q7WTB4	β-galactosidase	*Lactobacillus helveticus* (H)		−4.8	−5.5	−6.6	−7.7
Q1G9Z4	β-galactosidase	*Lactobacillus delbrueckii* subsp. *Bulgaricus* (H)		−7.0	−7.2	−8.3	−8.6
Q8X685	β-galactosidase	*Escherichia coli* O157:H7 (P)		−6.2	−6.7	−7.3	−7.4
A7KGA5	β-galactosidase	*Klebsiella pneumoniae* (P)		−4.6	−5.3	−6.1	−6.2

* Rows are grouped by enzyme class, i.e., by docking target: class A, arabinosidase-class enzymes (P49943, P45982, P42293); class B, β-galactosidase-class enzymes (A7LXS9, D4QFE7, A1A399, Q93GI5, O07013, O07012, Q7WTB4, Q1G9Z4, Q8X685, A7KGA5). The A and B labels denote these two enzyme groups and do not indicate that components A and B share binding-energy values; each enzyme was docked independently and each row reports that enzyme’s own binding energies. The first energy column is headed A/B because, in each row, it reports the component matched to that enzyme’s class: the arabinan (component B) in class-A rows and the galactan (component A) in class-B rows.

**Table 4:** Compound-specific half-saturation constants (K_1*POS*_, g/gXsu) derived from the binding energies and used for the CompuGUT simulation.

Component	Structure (DP)	K_1*POS*_ (g/gXsu) *
A	Galactan (DP2)	5.39 × 10^−3^
B	Arabinan (DP3)	1.59 × 10^−3^
C	Arabinogalactan (DP3)	2.51 × 10^−3^
D	Arabinogalactan (DP4)	8.63 × 10^−4^
E	Arabinogalactan (DP5)	3.97 × 10^−4^

* Average weighted half-saturation constants calculated from the binding energies toward the 13 glycosidases of Table 3, with weights reflecting the enzyme classes able to hydrolyze each component, so components A and B receive different values because their constants are averaged over different enzyme classes (Section 2.4.2 and Section 3.4.2).

## Data Availability

The original contributions presented in this study are included in the article/Supplementary Material. Further inquiries can be directed to the corresponding authors.

## Supplementary Materials

The following supporting information can be downloaded at: https://www.mdpi.com/article/10.3390/biom16101436/s1, Figure S1: Total ion chromatograms (TICs) of the five neutral oligosaccharide components (A–E) recorded during the UPLC-HILIC-MS/MS analysis of the P-1 fraction. The retention time (t_R_) of each component is indicated above the corresponding peak; Figure S2: Full-scan mass spectra (MS1) of components A–E, showing the precursor ions compiled in Table 2 of the main manuscript; Figure S3: Tandem mass spectra (MS/MS) of components A–E with the proposed fragment-ion assignments; diagnostic glycosidic-bond and cross-ring cleavage ions are labelled; Figure S4: Time-resolved CompuGUT simulations (28 days) of the colonic fermentation of components A–E in the intestinal lumen and in the mucosal layer (animated GIF files).

## Abbreviations

The following abbreviations are used in this manuscript: POS: pectin oligosaccharides; TFA: trifluoroacetic acid; PMP: 1-phenyl-3-methyl-5-pyrazolone; HPSEC: high-performance size-exclusion chromatography; RID: refractive index detector; UPLC-HILIC-MS/MS: ultra-performance liquid chromatography coupled to tandem mass spectrometry; DEAE: diethylaminoethyl; DP: degree of polymerization; HG: homogalacturonan; RG-I: rhamnogalacturonan I; RG-II: rhamnogalacturonan II; GH: glycoside hydrolase; CAZy: carbohydrate-active enzymes; SCFA: short-chain fatty acid; GalA: galacturonic acid.
