# Supplementary material for: Preparation, Structural Elucidation, and In Silico Prebiotic Evaluation of Pectin Oligosaccharides from Apple Pectin
Source: Biomolecules. 2026 Oct 2;16(10):1436. doi: 10.3390/biom16101436 (PMC13702196; doi:10.3390/biom16101436)

Figure S1. Total ion chromatograms (TICs) of the five neutral oligosaccharide components (A-E) recorded during the UPLC-HILIC-MS/MS analysis of the P-1 fraction. The retention time (t_R) of each component is indicated above the corresponding peak.

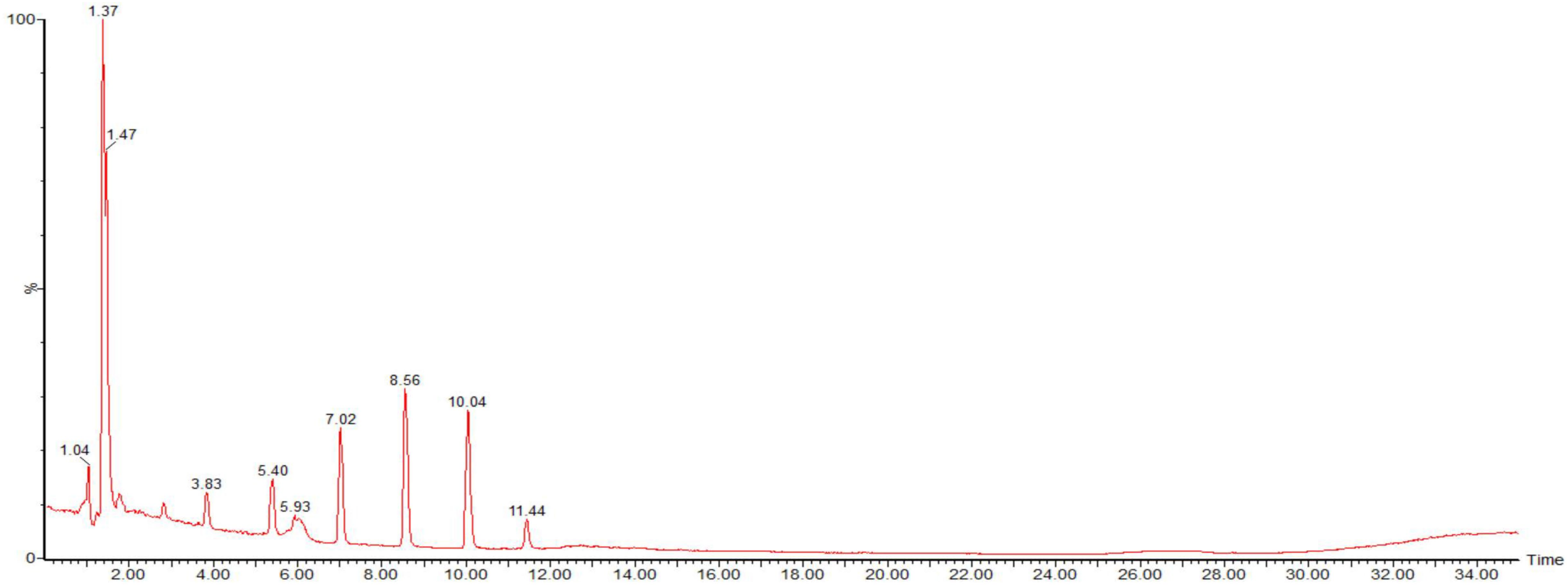

Figure S2. Full-scan mass spectra (MS1) of components A-E, showing the precursor ions compiled in Table 2 of the main manuscript.

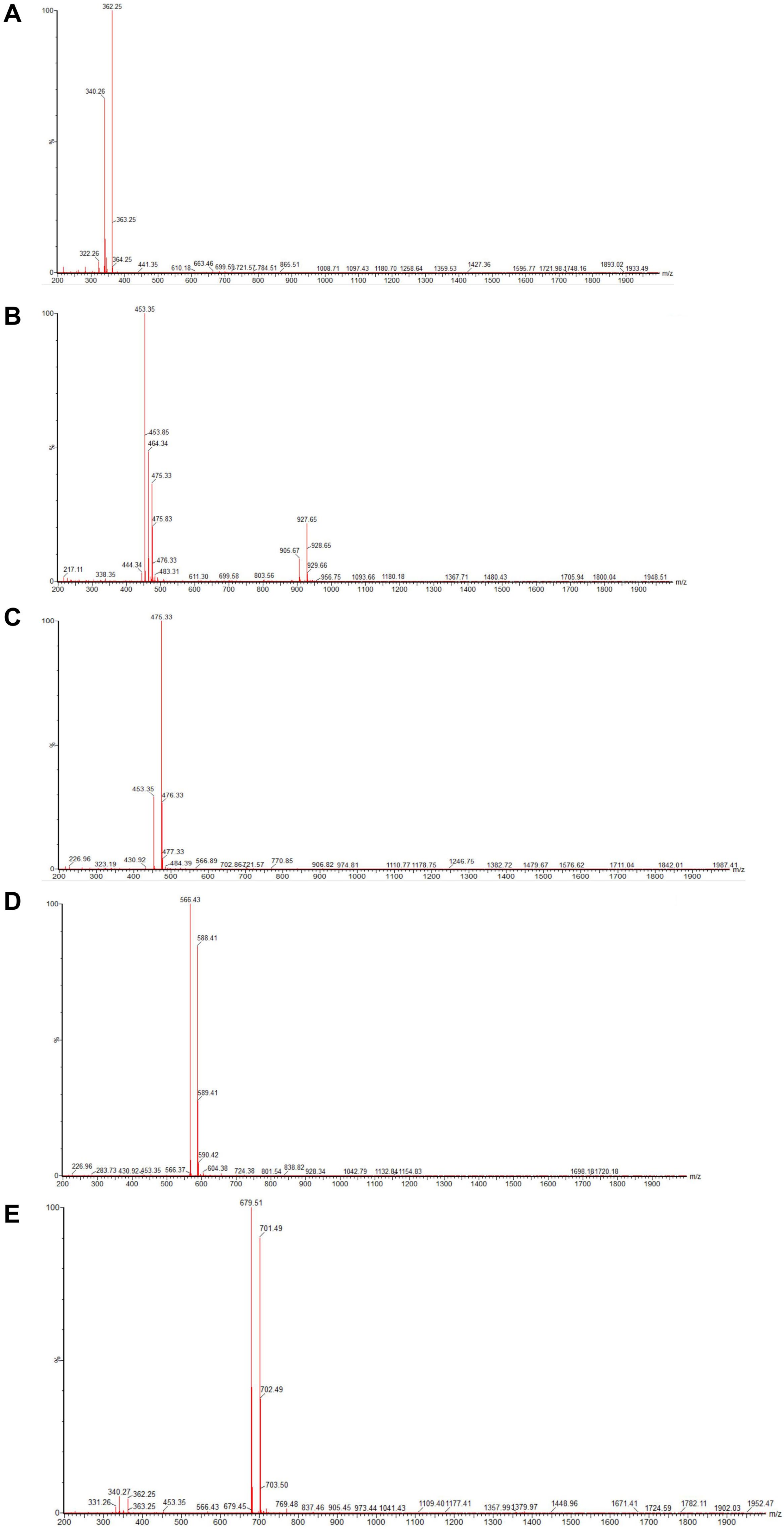

Figure S3. Tandem mass spectra (MS/MS) of components A–E with the proposed fragment-ion assignments; diagnostic glycosidic-bond and cross-ring cleavage ions are labelled.

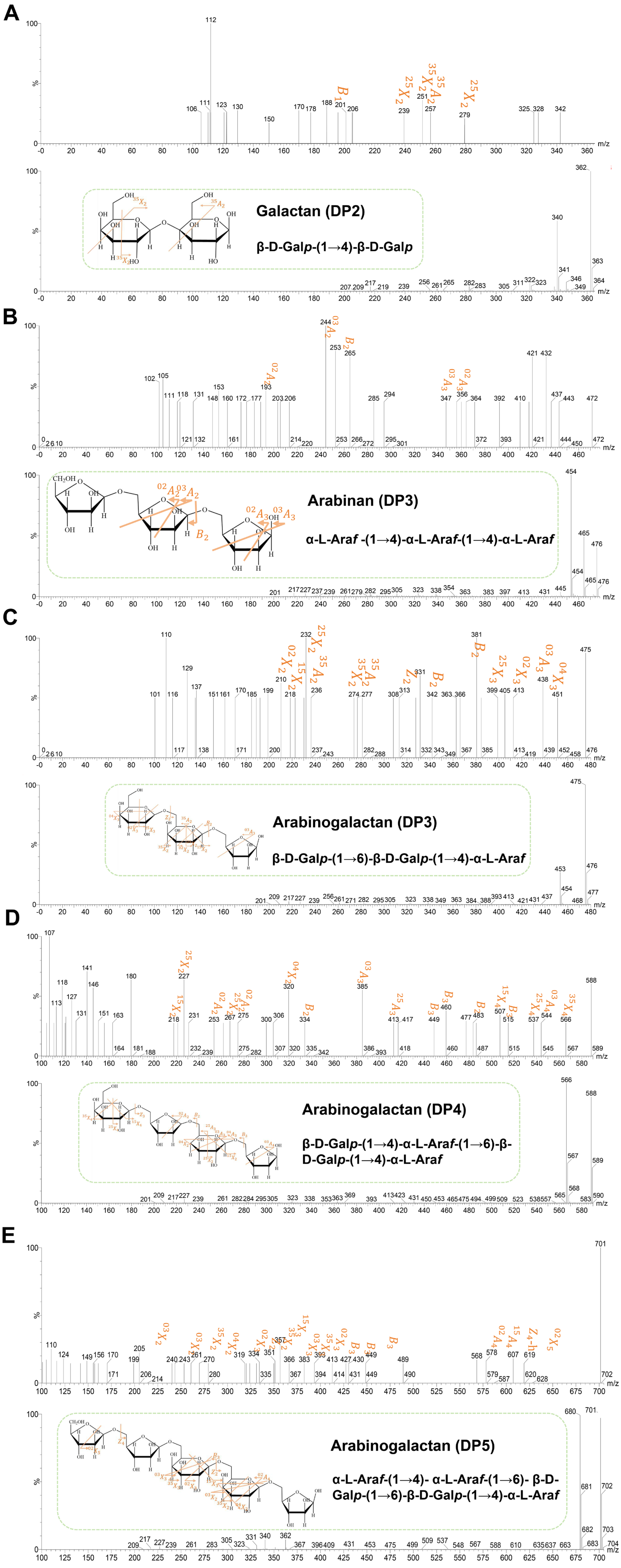

Supplement: Supplementary file 1 [file biomolecules-16-01436-s001.zip › Figure S1-S3.pdf]
